# Case report on anti-GQ1b antibody syndrome: initial symptoms of pupil palsy and periorbital pain

**DOI:** 10.3389/fimmu.2024.1474354

**Published:** 2024-12-17

**Authors:** Yang Du, Weidong Wang, Lili Zhang, Yuan Li, Xiang Chen, Hui Yang, Xin Ding

**Affiliations:** ^1^ Department of Neurology, Chengdu Second People's Hospital, Affiliated Hospital of Chengdu Medical College, Chengdu, Sichuan, China; ^2^ School of Clinical Medicine, Chengdu Medical College, Chengdu, Sichuan, China; ^3^ Department of Neurology, First AffiIiated Hospital of Chengdu Medical College, Chengdu, Sichuan, China

**Keywords:** GQ1b, GQ1b antibody, anti-GQ1b antibody syndrome, Miller Fischer syndrome, mydriasis, pain, opthalmoplegia

## Abstract

Anti-GQ1b antibody syndrome is a spectrum of autoimmune disorders affecting nervous systems. We report a case of a 53-year-old woman presenting mydriasis with acute onset of periorbital pain, photophobia, and subsequently, diplopia. Despite weakly positive anti-GQ1b IgG antibody, the patient exhibited atypical features with isolated ophthalmoplegia and absence of classic Miller-Fisher syndrome triad. Symptoms improved spontaneously without specific immunotherapy. This case highlights the variable clinical presentations of anti-GQ1b antibody syndrome, emphasizing the importance of considering this diagnosis in patients with unexplained iris abnormalities and ophthalmoplegia.

## Introduction

Anti-GQ1b antibody syndrome is a spectrum of autoimmune disorders affecting both the central and peripheral nervous systems. It encompasses Miller-Fisher syndrome (MFS), Guillain-Barré syndrome with ophthalmoplegia, Bickerstaff brainstem encephalitis, and acute ophthalmoplegia without ataxia (AO). The etiology is closely linked to the production of anti-GQ1b antibodies following infections with microorganisms such as Campylobacter jejuni and Haemophilus influenzae. Clinical manifestations are diverse and can include ophthalmoplegia, ataxia, areflexia, vestibular dysfunction, and optic neuropathy (1).

Iris paralysis is one of the hallmark features of anti-GQ1b antibody syndrome. Between 42% and 55% of patients with anti-GQ1b antibody syndrome exhibit iris paralysis, characterized by mydriasis, loss of light reflex, and anisocoria (2, 3).

Our patient’s initial presentation was characterized by mydriasis and orbital pain, which progressed to include diplopia. In the absence of other neurological manifestations, and supported by diagnostic workup, a diagnosis of AO syndrome was considered.

## Case report

A 53-year-old previously healthy Asian Chinese woman presented to the ophthalmology clinic with a one-day history of acute onset of persistent periorbital pain and photophobia. The patient denied any history of migraine, Graves’ disease, diabetes mellitus, syphilis, multiple sclerosis, neuromyelitis optica, recent trauma, or anticholinergic drug use. Two weeks ago, she reported a history of cough and fever, despite a negative test for Mycoplasma pneumoniae, which has been prevalent in her area. Initial examination revealed corrected visual acuity of 1.0/1.0 in both eyes, normal color vision, no conjunctival injection, and bilateral pupil size of 6mm with absent light and near reflexes. Ocular motility was normal with no diplopia. Slit-lamp and fundus examinations showed clear corneas, negative for keratic precipitates, no iris transillumination defects, pupillary displacement, or iris neovascularization. Visual evoked potentials (VEP) were normal in both eyes with normal latency and amplitude. Intraocular pressure was 16mmHg in the right eye and 15mmHg in the left eye. Coordination and deep tendon reflexes were normal.

Three days later, the patient’s periorbital pain and photophobia worsened, and she developed diplopia. Oral nonsteroidal anti-inflammatory drugs (lornoxicam 60mg) were given for pain relief. The patient was further admitted to the neurology department for treatment. Upon re-examination, visual acuity and color vision remained unchanged. Bilateral pupil size was 6mm, with absent light and near reflexes. Right eye adduction and infraduction were limited, with no scleral show. Slit-lamp and fundus examinations were normal. Coordination was normal on bilateral finger-nose and heel-shin tests, and gait, deep tendon reflexes, and other neurological examinations were also normal. Given the patient’s history of prodromal infection, serum antibody tests were performed to evaluate for central and peripheral demyelination. Western blot was used to assess peripheral demyelination, and indirect immunofluorescence was employed to evaluate central demyelination.

Five days later, the patient’s periorbital pain, photophobia, and diplopia persisted, prompting an adjustment to the treatment regimen. Intravenous dexamethasone (15mg) was initiated to alleviate pain. All laboratory tests, including complete blood count, C-reactive protein, calcitonin, HIV, and syphilis serology, were within normal limits. TORCH (Toxoplasma gondii, Rubella virus, Cytomegalovirus, and Herpes simplex virus) IgM was negative, ruling out recent infection. Chest CT did not reveal any evidence of inflammation. Lumbar puncture showed an opening pressure of 160mmHg H2O, cerebrospinal fluid (CSF) white blood cell count of 12.8*10^6/L (reference 0-8*10^6/L), protein of 333mg/L (reference 150-450mg/L), glucose of 3.2mmol/L (reference 2.5-4.4mmol/L), and chloride of 131mmol/L (reference 120-132mmol/L). CSF culture and Gram stain were negative. Serum anti-GQ1b IgG antibody was weakly postitive; anti-GQ1b IgM antibody was negative (as the **Supplementary Material**) anti-AQP4 IgG antibody was negative. Sensory, motor, and F-wave conduction of peripheral nerves of the extremities were normal. Brain, orbital, and intracranial vessel MRI scans were normal. Due to the possibility of blood-brain barrier damage caused by a slight elevation of white blood cells in the CSF, and the risk of further deterioration, we discussed the treatment option of intravenous immunoglobulin (IVIG). However, given the weakly positive serum anti-GQ1b IgG antibody, the absence of a typical Miller-Fisher syndrome (ophthalmoplegia, areflexia, ataxia), and the lack of supporting clinical studies, we did not administer IVIG.

**Figure 1:**
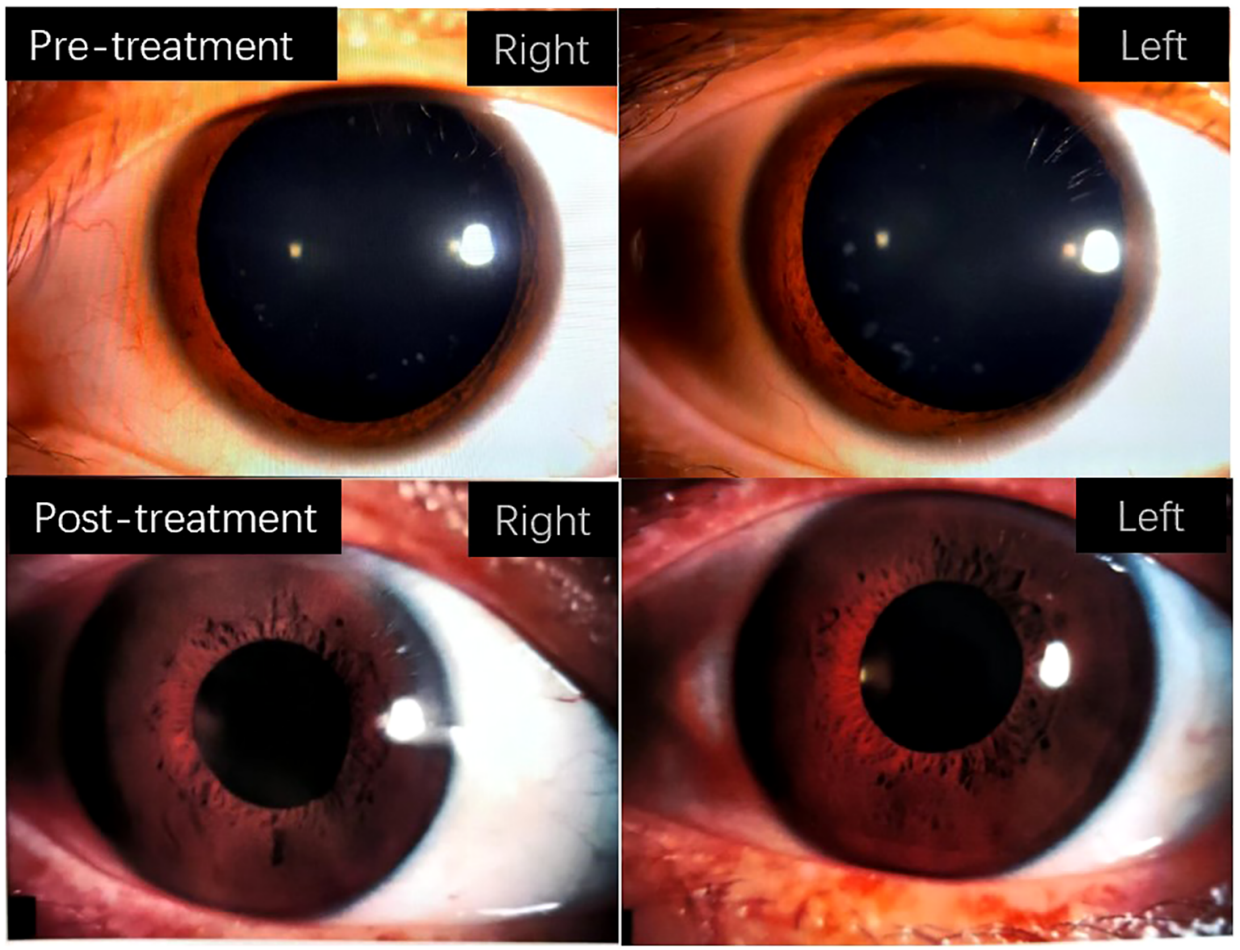
The figure depicts the changes in bilateral pupil size in patients before and after treatment.

**Figure 2:**
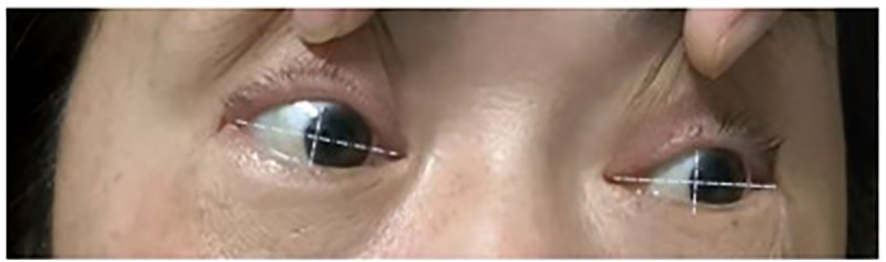
Physical examination revealed restricted adduction of the right eye compared to the left.

Ten days later, the patient’s periorbital pain and photophobia improved significantly, but diplopia persisted. Dexamethasone intravenous drip was discontinued. Further examination revealed a right pupil size of 3.5mm with sluggish light reflex, and a left pupil size of 3mm with brisk light reflex, as **Figure 1**. Diplopia was noted on examination, with restricted adduction and infraduction of the right eye, suggestive of a partial right oculomotor nerve palsy, as **Figure 2**. Coordination, gait, and deep tendon reflexes were normal. As the patient’s diplopia did not improve, and considering that immunomodulatory therapy can neutralize autoantibodies and inhibit complement activation in anti-GQ1b antibody syndrome, thus preventing subsequent pathophysiological effects, we still recommended IVIG therapy. However, due to economic reasons, the patient declined.

42 days later, a follow-up examination including slit-lamp examination revealed normal findings. The patient reported no recurrence of symptoms and was satisfied with the treatment outcomes.

## Discussion

The patient presented with a constellation of symptoms including periorbital pain, mydriasis, and restricted ocular motility. The limited and atypical nature of these symptoms posed a diagnostic challenge. After ruling out medications, trauma, vascular causes, iritis, angle-closure glaucoma, myasthenia gravis, and specific medical history, despite normal cerebrospinal fluid protein and glucose levels, the preceding infection inclined us towards a diagnosis of a neurological infection or an immune-mediated disorder. Previous reports have documented similar clinical manifestations in botulism type B (4), associated with the ingestion of improperly preserved food, and West Nile virus infection (5), prevalent in Africa and North America and transmitted by mosquito bites. However, the patient denied any relevant exposure history. Similarly, neurotropic viruses such as dengue and Zika can cause analogous symptoms, although these diseases are not endemic to the patient’s region. The diagnosis of anti-GQ1b antibody syndrome was established based on the absence of AQP4 antibody which are specific markers for neuromyelitis optica spectrum disorders (6) and the presence of anti-GQ1b antibody.

GQ1b ganglioside is highly concentrated in cranial nerves III, IV, and VI, the central nervous system, muscle spindles of the limbs, and the cerebellum (7–9). By influencing neurogenesis, neurotransmission, and axonal growth, it plays a role in neural plasticity and is associated with many autoimmune peripheral neuropathies. GQ1b ganglioside is widely distributed throughout the central and peripheral nervous systems and can lead to various clinical manifestations. These may include ophthalmoplegia, ataxia, areflexia, vestibular dysfunction, and optic neuropathy.

Some patients with anti-GQ1b antibodies may present with acute isolated ophthalmoplegia (AO), without areflexia or ataxia (2, 10, 11). Neurophysiological studies and CSF analysis in patients with AO-type anti-GQ1b antibody syndrome are often unremarkable (2, 12).Therefore, ocular motility and pupillary changes are crucial clinical signs in diagnosing this condition before antibody test results are available.

Previous reports of ophthalmoplegia associated with anti-GQ1b antibody syndrome typically involve bilateral involvement, with 27% of cases presenting with asymmetric or unilateral involvement, and the abducens nerve being relatively commonly affected (13, 14). In contrast, our patient presented with unilateral partial oculomotor nerve palsy. Previous studies have reported pupillary involvement in 6 out of 34 patients with anti-GQ1b antibody syndrome. The manifestations of pupillary involvement included fixed dilated pupils, tonic pupils, and anisocoria (2, 3). Abigail et al. (15) described a case with an initial presentation similar to our patient, characterized by bilateral mydriasis and headache, but subsequently developed bilateral lateral rectus palsy and balance disorder. In contrast, our patient presented with isolated partial oculomotor nerve palsy, which is less commonly reported.

Pain is not a common symptom in anti-GQ1b antibody syndrome. In a retrospective study (16), only 3 out of the patients with anti-GQ1b antibodies expressed different forms of pain. Some reports (17) suggest that headache may be associated with benign intracranial hypertension due to impaired CSF outflow caused by elevated protein levels, while others (18) propose that trigeminal and upper spinal root damage mediated by anti-GQ1b antibodies activates the trigemino-vascular pain pathway. Additionally, David et al. (19) reported a case with bilateral pulsating pain accompanied by bilateral abducens nerve palsy, suggesting a potential correlation between the two. In our patient, given the normal CSF and ocular pressure, the absence of significant neuroinflammatory changes on MRI, and given the absence of abducens nerve palsy, we tend to favor the possibility of anti-GQ1b antibody-mediated trigeminal ophthalmic branch damage. The underlying mechanisms of pain in anti-GQ1b antibody syndrome still require further investigation.

Referencing the treatment of MFS (20), IVIG remains the first-line treatment, and previous clinical studies have demonstrated that IVIG can accelerate patient recovery (21). In contrast, our patient showed significant improvement with only non-steroidal anti-inflammatory drugs and low-dose corticosteroids. This may be related to the patient’s weakly positive anti-GQ1b IgG antibody titer and milder neurological involvement, consistent with previous findings (7). Previous studies (22) have demonstrated that dexamethasone can activate glucocorticoid receptors, leading to the expression of dynorphin A, which contributes to an antihyperalgesic effect in neuropathic pain, thereby alleviating neuropathic pain. Additionally, low-dose glucocorticoids do not exhibit immunomodulatory effects (11). Based on this, we propose that low-dose glucocorticoid use does not influence disease progression. This further supports the notion that the condition may be self-limiting when neurological involvement is minimal. Larger studies are still needed to determine the optimal treatment for anti-GQ1b antibody syndrome. Previous studies have shown that ophthalmoplegia caused by anti-GQ1b antibody syndrome often fully recovers within 29-165 days (3). Our patient, however, achieved complete resolution of ophthalmoplegia on day 42 of the disease course.

## Conclusions

The clinical manifestations of anti-GQ1b antibody syndrome spectrum disorders are diverse. Focusing on non-specific symptoms such as pupillary changes, ocular movement abnormalities, and pain is crucial for early identification.

## Data Availability

The raw data supporting the conclusions of this article will be made available by the authors, without undue reservation.
