# Supplementary material for: Case report on anti-GQ1b antibody syndrome: initial symptoms of pupil palsy and periorbital pain
Source: Front Immunol. 2024 Dec 17;15:1474354. doi: 10.3389/fimmu.2024.1474354 (PMC11685045; doi:10.3389/fimmu.2024.1474354)
Supplement: Supplementary file 1 [file Image1.pdf]

Antibody Tests of Peripheral Demyelination

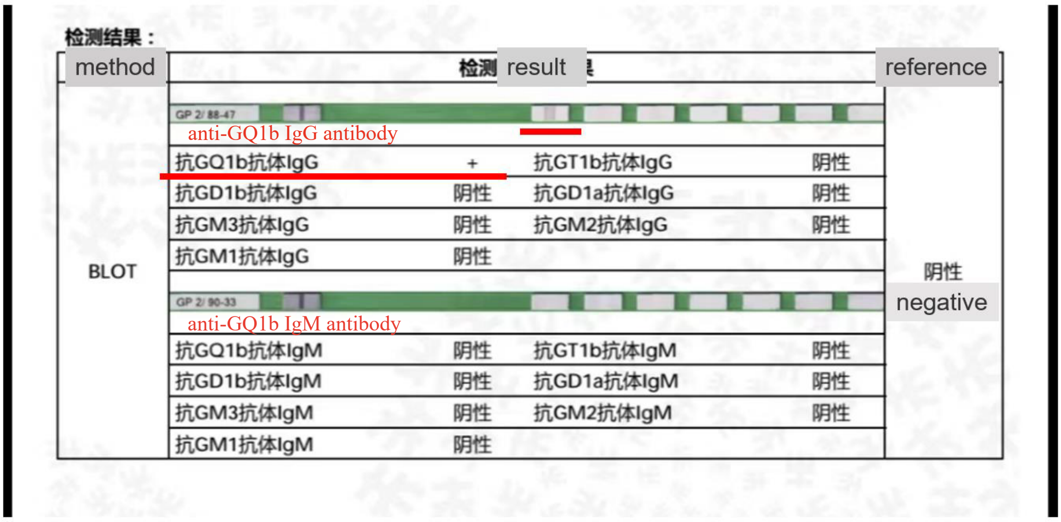

检测结果：

method	检测 result				reference
BLOT	GP 2/ 88-47				阴性 negative
	anti-GQ1b IgG antibody				
	抗GQ1b抗体IgG	+	抗GT1b抗体IgG	阴性	
	抗GD1b抗体IgG	阴性	抗GD1a抗体IgG	阴性	
	抗GM3抗体IgG	阴性	抗GM2抗体IgG	阴性	
	抗GM1抗体IgG	阴性			
	GP 2/ 90-33				
	anti-GQ1b IgM antibody				
	抗GQ1b抗体IgM	阴性	抗GT1b抗体IgM	阴性	
	抗GD1b抗体IgM	阴性	抗GD1a抗体IgM	阴性	
	抗GM3抗体IgM	阴性	抗GM2抗体IgM	阴性	
	抗GM1抗体IgM	阴性			

Serum anti-GQ1b IgG antibody was weakly postitive; anti-GQ1b IgM antibody was negative.
